# Analysis of the correlation between the serum residual cholesterol level at admission and the risk of death after discharge in patients with ischemic stroke

**DOI:** 10.5937/jomb0-59233

**Published:** 2026-01-28

**Authors:** De Xu, Ruijuan Duan, Ruiqi Zhu, Yinghua Huang, Shiyu Chen

**Affiliations:** 1 Jinhua People's Hospital, Department of Neurology, Jinhua City, China; 2 Peking University Shenzhen Hospital, Department of Neurology, Shenzhen City, China

**Keywords:** ischemic stroke, blood lipid level, residual cholesterol, risk of death, serum residual cholesterol level, ishemijski moždani udar, nivo lipida u krvi, rezidualni holesterol, rizik od smrti, rezidualni holesterol u serumu

## Abstract

**Background:**

To investigate the connection betweenischemic stroke (IS) patients' risk of dying after being discharged and their residual cholesterol (RC) levels uponadmission.

**Methods:**

2021 IS patients between the ages of 35 and 80were chosen as the study's subjects, and data on deathendpoints following discharge were gathered. The doseresponse association between the risk of death and the RCat admission was examined using restricted cubic spline(RCS) regression. The hazard ratio (HR) and 95% CI werecalculated via Cox regression to analyse the associationbetween the RC level at admission and the risk of deathafter discharge in patients with IS.

**Results:**

According to the RCS model, RC levels were nonlinearly associated with deaths from IS and other causes(P<0.001). With the median RC level as the cutoff value,the subjects were divided into two groups: a low RC group(RC<0.72 mmol/L) and a high RC group (RC≥0.72mmol/L). Compared with those in the high RC group, theage and male ratio in the low RC group were significantlygreater. The fasting blood glucose (GLU), total cholesterol(TC), triglyceride (TG), low-density lipoprotein cholesterol(LDL-C), non-high-density lipoprotein cholesterol (nonHDL-C), apolipoprotein A-1 (ApoA-1), and apolipoproteinB (ApoB) levels, as well as diabetes rates, were lower (P=0.01). Cox regression analysis revealed that withoutadjusting for covariates, the high-level RC group presenteda lower risk of all-cause death than the low-level RC group(HR=0.765, 95% CI: 0.619~0.946, P=0.013) and alower risk of death from IS (HR = 0.638, 95% CI:0.435~0.936, P=0.022). After adjusting for sex, age,smoking status, drinking status, hypertension status, anddiabetes status, the high-level group still had a lower risk ofall-cause death (HR = 760, 95% CI: 0.614~0.941,P=0.012) and a lower risk of death from IS (HR=0.653,95% CI: 0.444-0.961, P=0.031). Male sex (HR=0.753,95% CI: 0.572~0.990, P=0.042). Age ≥65 years (HR=0.598, 95% CI: 0.391~0.916, P=0.018), nonsmokingstatus (HR=0.628, 95% CI: 0.408~0.967, P=0.035),nonalcoholic status (HR=0.656, 95% CI: 0.439~0.979,P=0.039), not complicated with hypertension (HR=0.321, 95% CI: 0.108~0.957, P=0.041), no diabetesmellitus (HR=0.607, 95% CI: 0.389~0.947, P=0.028).Compared with those in the high RC group, the IS patientsin the low RC group had a lower incidence of all-causedeath, IS death and other causes of death and a higher survival rate.

**Conclusions:**

An RC<0.72 mmol/L at admission is associated with an increased risk of all-cause death and longterm IS death after discharge.

## Abstract (sr)

**Uvod:**

Cilj je bio da se ispita veza između rizika od smrtinakon otpusta kod pacijenata sa ishemijskim moždanimudarom (IMU) i nivoa rezidualnog holesterola (RH) u serumu pri prijemu.

**Metode:**

Za studiju je izabrano 2021 pacijenata sa IMU,starosti između 35 i 80 godina. Takođe su prikupljeni ipodaci o smrtnim ishodima nakon otpusta. Odnos doze iodgovora između rizika od smrti i nivoa RH pri prijemu jeanaliziran korišćenjem regresije sa ograničenim kubnimsplajnovima (RCS). Regresijom po Koksovom modelu suizračunati hazardni odnosi (HR) i 95% intervali puzdanosti(CI) radi analize povezanosti nivoa RH pri prijemu i rizika odsmrti nakon otpusta kod pacijenata sa IMU.

**Rezultati:**

Prema RCS modelu, nivo RH je bio nelinearnopovezan sa smrću usled IMU i drugih uzroka (P<0,001). Sa medijanom RH kao graničnom vrednošću, ispitanici supodeljeni u dve grupe: grupa sa niskim RH (RH<0,72mmol/L) i grupa sa visokim RH (RH≥0,72 mmol/L). Upoređenju sa grupom sa visokim RH, grupa sa niskim RHje imala značajno veći prosek godina i udeo muškaraca.Nivoi glukoze u krvi natašte (GLU), ukupnog holesterola(TC), triglicerida (TG), holesterola lipoproteina niske gustine (LDL-C), ne-HDL holesterola (non-HDL-C), apolipo -proteina A-1 (ApoA-1) i apolipoproteina B (ApoB), kao istopa dijabetesa, bili su niži (P=0,01). Koksova regresiona analiza pokazala je da, bez podesavanja za kovarijate,grupa sa visokim RH ima manji rizik od smrti iz svih uzrokau poređenju sa grupom sa niskim RH (HR=0,765, 95% CI:0,619-0,946, P=0,013), kao i manji rizik od smrti usledIMU (H R=0,638, 95% CI: 0,435-0,936, P=0,022).Nakon podesavanja za pol, starost, pusenje, konzumacijualkohola, hipertenziju i dijabetes, grupa sa visokim RH idalje je imala manji rizik od smrti iz svih uzroka (HR=0,760, 95% CI: 0,614-0,941, P=0,012) i manji rizik odsmrti usled IMU (HR=0,653, 95% CI: 0,444-0,961,P=0,031). Faktori povezani sa nižim rizikom od smrtiuključuju muski pol (HR=0,753, 95% CI: 0,572~0,990,P=0,042), starost ≥65 godina (HR=0,598, 95% CI:0,391~0,916, P=0,018), nepusenje (HR=0,628, 95%CI: 0,408-0,967, P=0,035), nekonzumiranje alkohola(HR= 0,656, 95% CI: 0,439~0,979, P=0,039), odsustvohipertenzije (HR=0,321, 95% CI: 0,108~0,957, P=0,041)i odsustvo dijabetesa (HR=0,607, 95% CI: 0,389~0,947,P=0,028). U poređenju sa grupom sa visokim RH, pacijenti sa IMU iz grupe sa niskim RH imali su nižu stopu smrtiiz svih uzroka, nižu stopu smrti usled IMU i drugih uzroka,kao i visu stopu preživljavanja.

**Zaključak:**

Nivo RH<0,72 mmol/L pri prijemu povezan jesa povecanim rizikom od smrti od svih uzroka i kasnije smrtiusled IMU nakon otpusta.

## Introduction

As the most common type of cardiovascular and cerebrovascular disease, stroke is characterised by high morbidity, disability and recurrence rates. It has become the top priority for medical and healthcare worldwide [1] [2] [3]. To protect people's lives and health, it is urgent to combine tertiary prevention and strive to improve patient prognosis while further strengthening prevention and treatment.

Ischemic stroke (IS) is influenced by several risk factors, including dyslipidemia, smoking, alcohol consumption, diabetes, hypertension, heart defects, psychosocial factors, and lifestyle factors [4] [5] [6]. Among these factors, dyslipidemia is a significant risk factor that can be modified. By 2012, the prevalence rate of dyslipidemia in Chinese residents aged 18 years and above reached 40.4%, showing an overall increasing trend [7]. Dyslipidemia is a risk factor for the occurrence and adverse outcomes of IS, and further study is urgently needed. A series of studies on blood lipid indices and the occurrence of IS have shown that total cholesterol (TC), triglyceride (TG) and low-density lipoprotein cholesterol (LDL-C) are risk factors for the occurrence and recurrence of IS, whereas high-density lipoprotein cholesterol (HDL-C) is a protective factor [8] [9] [10]. Elevated levels of lipoprotein a [Lp(a)] and apolipoprotein B (ApoB) are risk factors for the development of IS, whereas the role of apolipoprotein A-1 (ApoA-1) is significantly weakened after correlation adjustment. With respect to the relationship between lipid indices and the prognosis of IS, high, low and fluctuating TC levels can lead to the recurrence of IS, poor short-term prognosis and long-term death. Hypertension is a protective factor for the short-term prognosis of IS, and lower TG levels are associated with poorer NIHSS and mRS indices and 3-month mortality in IS patients [11] [12] [13] [14]. This study revealed that lower TG levels at admission (1.3 mmol/L) were associated with increased mortality. Using drugs to maintain patients with low LDL-C levels can reduce the occurrence and development of cerebrovascular events, and can effectively reduce the recurrence rate of stroke. In contrast, high HDL-C levels can reduce the possibility of neurological defects after thrombolytic therapy [15]. With increasing research, the effect of residual cholesterol (RC) on IS has gradually become a hot topic in recent years. Currently, academic circles have not established uniform reference values for RC. Previous studies [16] [17] [18] have shown that the higher the RC level, the greater the risk of IS in the population, and lower RC levels reduce the recurrence rate of cardiovascular events after IS by approximately 20%. However, studies on the association between RC and IS prognosis have just started, the sample sizes of relevant studies on IS are small, and the follow-up time is short; thus, more studies are needed to explore the relationship between RC and IS prognosis. This study investigated the relationships between the RC level at admission and the risk of death after discharge in IS patients and between the IS, the dose response between the RC and the risk of death in IS patients, and the relation ship with death prognosis in IS patients to provide a scientific basis and accurate guidance for tertiary prevention of IS.

## Materials and methods

### Research subjects

A unified questionnaire was developed to collect data from 2021 IS patients admitted to our hospital between April 8, 2018, and December 25, 2024.

Inclusion criteria: (1) Fulfilled the International Guidelines for the Diagnosis and Treatment of Acute Ischemic Stroke's IS diagnostic requirements; (2) Aged 35-80 years.

Exclusion criteria: (1) Incomplete case data; (2) A definite history of autoimmune disease or tumour; (3) Severe liver or kidney disease.

### Collection of clinical data

Smoking history was defined as 20 cigarettes/day for more than 10 years. A history of alcohol consumption was defined as 3 drinks/day for more than 10 years. Diabetes is defined as a fasting blood glucose (FBG) level ≥7.0 mmol/L (126 mg/dL), a self reported physician diagnosis, or the use of diabetes medications.

### Detection of blood indicators

At admission, fasting levels of GLU, TC, TG, HDL-C, LDL-C, ApoA-1, ApoB, and Lp(a) were detected in all participants. RC, non-HDL-C (non-HDL-C), TC/HDL-C, TG/HDL-C, and LDL-C/HDL-C were calculated.

### Follow-up investigation method

Patients were followed up centrally from December 2023 to April 2024, with admission diagnosis as the starting point for IS and termination of follow-up or death as the end point, and outcomes, including all-cause death, stroke death, IS death, hemorrhagic stroke (HS) death and other causes of death, were recorded.

### Statistical methods

The ratio or composition of the data was expressed, and the 2 test was used to compare the data between groups. The subjects were divided into two groups, a low RC group and a high RC group, based on the median RC level. The baseline charac teristics of the population and between-group differences were analysed using ANOVA or non-parametric tests. A restricted cubic spline (RCS) regression analysis was performed to describe the linear or nonlinear correlation between the RC level and the risk of death with different prognoses in patients with IS. The cutoff value of the RC index was determined, and the subjects were grouped according to the cutoff value combined with the RC level at admission. Univariate Cox regression analysis did not adjust for covariates. Multivariate Cox regression analysis adjusted for covariates. A Kaplan Meier curve was used to plot the survival curves of patients with baseline RC levels and different causes of death. Both sides' P values were less than 0.05, indicating a statistically significant difference.

## Results

### Follow-up outcomes

The median follow-up was 5.52 years. All-cause death occurred in 2021 patients, 168 of whom died from stroke (114 from IS, 54 from HS) and 191 from other causes.

### RC and various outcomes were fitted using a restricted cubic spline regression model

**Figure 1:**
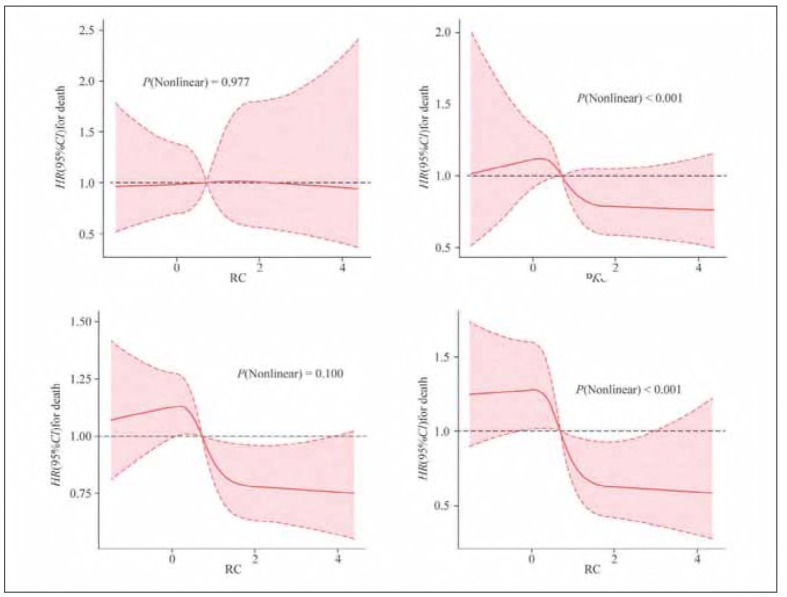
RCS regression analysis of RC level at admission and risk of death with IS prognosis.

The RCS regression results take the median RC level as the reference line of HR=1 and combine the RCS regression curve relationship, showing that when the RC level is less than the median, HR<1; when the RC level is greater than or equal to the median, HR is greater than or equal to 1, so the median RC water level (0.72 mmol/L) is selected as the cutoff value. According to the RC median, the subjects were divided into 1,012 cases in the low-level RC group (RC<0.72 mmol/L) and 1,009 cases in the high-level RC group (RC≥0.72 mmol/L) (Figure 1).

### RCS regression analysis of patients' RC level and prognostic risk of all-cause death at admission

RC levels were nonlinearly associated with the risk of all-cause death (P=0.01) and nonlinearly associated with death from other causes (P<0.01). When the RC level was <0.72 mmol/L. When the RC level was close to 0.72 mmol/L, the risk of death increased with the RC level, then rapidly decreased for a short time, and finally decreased steadily.

### RCS regression analysis of the RC level at admission and risk of death with a prognostic IS

A significant nonlinear correlation was found between the RC level and the risk of IS mortality (P<0.001). When the RC level was <0.72 mmol/L, the IS mortality risk showed a relatively stable trend. When the RC water level was close to 0.72 mmol/L, the risk of IS death decreased rapidly for a short time with increasing RC level and then steadily declined.

### RCS regression analysis of the RC level at admission and the prognostic risk of HS death

The RCS curve for the death outcome of HS death was described, and the RC level was not linearly correlated with the risk of HS death (P=0.977). With increasing RC level, the death risk of HS always showed a relatively stable trend without significant fluctuations.

### RCS regression analysis of RC level at admission and risk of death from other causes

There was a significant nonlinear association between the RC level and death from other causes (P<0.001). When the RC level was <0.72 mmol/L, as the RC level rose, so did the danger of death. When the RC level approached 0.72 mmol/L, as the RC level rose, so did the threat of death; however, the increasing RC level rapidly decreased for a short time and then steadily decreased.

### Comparison of clinical data between the low-level RC group and the high-level RC group

Compared with those in the high RC group, the age and male ratio in the low RC group were significantly greater. GLU, TC, TG, LDL-C, non-HDL-C, ApoA-1, ApoB, water, TG/HDL-C, TG/HDL-C, LDL-C/HDL-C, and the diabetes rate were significantly lower (P<0.05~0.01) (Table 1).

**Table 1:** Comparison of clinical data between low and high level RC group [Cases (%), M (Q 5, Q s)].

Group	Number of cases	Age (Years)	Male	Smoking history	Drinking history	History of diabetes	History of hypertension	SBP (mmHg)
Low-level RC group	1012	67.00 (59.88, 73.00)	658 (65.02)	264 (26.09)	159 (15.71)	246 (24.31)	715 (70.65)	150.00 (137.00, 160.00)
High-level RC group	1009	(59.66, 70.00)	552 (54.71)	235 (23.29)	164 (16.25)	315 (31.22)	750 (74.33)	150.00 (136.00, 162.00)
P value		0.032	<0.001	0.160	0.786	0.001	0.072	0.156
Group	Number of cases	DBP (mmHg)	GLU (mmol/L)	TC (mmol/L)	TG (mmol/L)	HDL-C (mmol/L)	LDL-C (mmol/L)	RC (mmol/L)
Low-level RC group	1012	90.00 (80.00, 96.25)	4.52 (4.24, 5.15)	4.11 (3.55, 4.69)	1.14 (0.80, 1.53)	1.14 (0.97, 1.35)	2.53 (2.03, 3.09)	0.45 (0.26, 0.58)
High-level RC group	1009	90.00 (80.00, 96.00)	5.08 (4.46, 5.48)	5.22 (4.53, 5.87)	1.78 (1.21, 2.66)	1.13 (0.94, 1.35)	2.80 (2.23, 3.31)	1.08 (0.87, 1.45)
P value		0.187	<0.001	<0.001	<0.001	0.098	<0.001	<0.001
Group	Number of cases	Non-HDL-C (mmol/L)	TC/HDL-C	TG/ HDL-C	LDL-C/ HDL-C	ApoA4 (g/L)	ApoB (g/L)	Lp (a) (mg/L)
Low-level RC group	1012	2.93 (2.39, 3.46)	3.55 (2.99, 4.12)	0.96 (0.65, 1.43)	2.20 (1.75, 2.72)	1.32 (1.17, 1.47)	0.85 (0.70, 1.00)	143.05 (85.35, 259.65)
High-level RC group	1009	4.03 (3.42, 4.63)	4.55 (3.86, 5.43)	1.56 (0.97, 2.52)	2.44 (1.92, 3.07)	1.37 (1.24, 1.53)	1.00 (0.85, 1.15)	143.40 (80.60, 258.50)
P value		<0.001	<0.001	<0.001	<0.001	<0.001	<0.001	0.719
				TOAST classification				
Group	Number of cases	Cardiac embolism (CE)	Large atherosclerosis type (LAA)	Arteriole occlusive or lacunar (SAO)	Other etiological types (SOE)	Unknown cause (SUE)		
Low-level RC group	1012	51 (5.04)	395 (39.03)	553 (54.64)	7 (0.69)	6 (0.59)		
High-level RC group	1009	38 (3.77)	416 (41.23)	548 (54.31)	3 (0.30)	4 (0.40)		
P value				0.347				

### Cox regression analysis of the associations between RC and different prognostic outcome events

Cox regression analysis was performed to examine death outcomes in relation to varying RC levels and ISs. Without adjusting for covariates, the high-level RC group presented a lower risk of all-cause death than the low-level RC group did (HR=0.765, 95% CI: 0.619~0.946, P=0.013) and a lower risk of death from IS (HR=0.638, 95% CI: 0.435~0.936, P=0.022) (Table 2).

**Table 2:** Cox regression analysis of RC level and IS prognostic death outcome. Note: ref. as the reference group

		All-cause death				
Group	Number of cases	Number of deaths	No adjusted covariates		Adjustment covariate	
			HR (95%C)	P value	HR (95%CD)	P value
Low-level RC group	1012	215	ref	-	ref	-
High-level RC group	1009	144	0.765 (0.619~0.946)	0.013	0.760 (0.614~0.941)	0.012
		Stroke death				
Group	Number of cases	Number of deaths	No adjusted covariates		Adjustment covariate	
			HR (95%CI)	P value	HR (95%CI)	P value
Low-level RC group	1012	100	ref	-	ref	-
High-level RC group	1009	68	0.748 (0.549~1.019)	0.066	0.769 (0.563~1.050)	0.099
		IS dead				
Group	Number of cases	Number of deaths	No adjusted covariates		Adjustment covariate	
			HR (95%CI)	P value	HR (95%CI)	P value
Low-level RC group	1012	72	ref	-	ref	-
High-level RC group	1009	42	0.638 (0.435~0.936)	0.022	0.653 (0.444~0.961)	0.031
		HS death				
Group	Number of cases	Number of deaths	No adjusted covariates		Adjustment covariate	
			HR (95%CI)	P value	HR (95%CI)	P value
Low-level RC group	1012	36	ref	-	ref	-
High-level RC group	1009	18	0.823 (0.446~1.519)	0.534	0.851 (0.459~1.578)	0.608
		Death from other causes				
Group	Number of cases	Number of deaths	No adjusted covariates		Adjustment covariate	
			HR (95%CI)	P value	HR (95%CI)	P value
Low-level RC group	1012	115	ref	-	ref	-
High-level RC group	1009	76	0.781 (0.584~1.044)	0.095	0.754 (0.562~1.010)	0.059

The risk of all-cause death was lower in the high RC group than in the low RC group even after con trolling for sex, age, smoking, alcohol use, hypertension, and diabetes (HR=760, 95% CI: 0.614~0.941, P=0.012). A lower risk of death from IS (HR=0.653, 95% CI: 0.444-0.961, P=0.031), death from diencephalic stroke in both groups (HR=0.769, 95% CI: 0.0563~1.050, P=0.099), HS deaths (HR=0.0851, 95% CI: 0.0459-1.578, P=0.0608) and other causes of death (HR=0.0754, 95% CI: 0.0851, 95% CI: 0.0459-1.578, P=0.0608). 562~1.010, P=0. The difference was not statistically significant (Table 2).

### Subgroup analysis by Cox regression

The patients were divided into different subgroups according to sex, age, smoking status, alcohol consumption status, hypertension status, diabetes history, dyslipidemia status and TOAST classification, and the baseline RC level and prognostic risk of all-cause death and IS death in the different subgroups were analysed. Correlation analysis revealed that male sex (HR=0.753, 95% CI: 0. 572~0. 990, P=0.042), age≥65 years (HR=0. 755, 95% CI: 0. 594~0. 959, P=0. 021), nonsmoking (HR=0. A total of 746, 95% CI: 0.590~0.943, P=0.014), nonalcohol consumption (HR=0.735, 95% CI: 0.588~0.919, P=0.007), hypertension (HR=0.738, 95% CI: 0.580~0.940, P=0.014), and no diabetes mellitus (HR=0.724, 95% CI: 0.561 0.934, P=0.013) were associated with a statistically significant reduction in the risk of all-cause death (Table 3).

**Table 3:** Analysis of association between subgroups with different RC levels and risk of all-cause death (unadjusted covariates). Note: ref. as the reference group

Factor	RC<0.72 mmol/L				RC≥0.72 mmol/L				
	Number of cases	Number of deaths	HR (95%CI)	P value	Number of cases	Number of deaths	HR (95%CD)		P value
Sex									
Male	658	147	ref.	-	552	79	0.753 (0.572~0.990)		0.042
Female	354	69	ref.	-	457	64	0.817 (0.581~1.149)		0.244
Age									
<65 years	417	39	ref.	-	451	34	0.944 (0.595~1.497)		0.806
≥ 65 years	595	177	ref.	-	558	109	0.755 (0.594~0.959)		0.021
Smoking									
No	748	172	ref.	-	774	119	0.746 (0.590~0.943)		0.014
Yes	264	44	ref.	-	235	24	0.818 (0.495~1.350)		0.432
Tipple									
No	853	197	ref.	-	845	127	0.735 (0.588~0.919)		0.007
Yes	159	19	ref.	-	164	16	1.042 (0.532~2.041)		0.904
Hypertension									
No	297	52	ref.	-	259	32	0.830 (0.533~1.291)		0.408
Yes	715	164	ref.	-	750	111	0.738 (0.580~0.940)		0.014
Diabetes									
No	766	162	ref.	-	694	94	0.724 (0.561~0.934)		0.013
Yes	246	54	ref.	-	315	49	0.836 (0.566~1.233)		0.366
Dyslipemia									
No	563	119	ref.	-	334	40	0.723 (0.446~1.171)		0.188
Yes	449	97	ref.	-	675	103	0.780 (0.606~1.005)		0.055
TOAST classification									
LAA	395	84	ref.	-	416	71	0.817 (0.581~1.149)		0.244
SAO	553	111	ref.	-	548	59	0.817 (0.581~1.149)		0.244
Other typing	64	21	ref.	-	45	13	0.817 (0.581~1.149)		0.244

There was a statistically significant decrease in the risk of dying from IS among those aged S65 years (HR=0.59, 95% CI: 0.391~0.916, P=0.018), not smoking (HR=0.628, 95% CI: 0.408~0.967, P = 0.035), not drinking (HR=0.0656, 95% CI: 0.0439~0.979, P=0.039), not having hypertension (HR=0.321, 95% CI: 0.108~0.957, P=0.041), and not having diabetes (HR=0.7795% CI: 0.389 0.947, P=0.028) (Table 4).

**Table 4:** Analysis of association between subgroups with different RC levels and risk of death from IS (unadjusted covariates). Note: ref. as the reference group

Factor	RC<0.72 mmol/L				RC≥0.72 mmol/L				
	Number of cases	Number of deaths	HR (95%CI)	P value	Number of cases	Number of deaths	HR (95%CI)		P value
Sex									
Male	658	51	ref.	-	552	26	0.699 (0.435-1.123)		0.139
Female	354	22	ref.	-	457	15	0.588 (0.305-1.135)		0.114
Factor	RC<0.72 mmol/L				RC≥0.72 mmol/L				
	Number of cases	Number of deaths	HR (95%CI)	P value	Number of cases	Number of deaths	HR (95%CI)		P value
Age									
<65 years	417	9	ref.	-	451	9	1.111 (0.440-2.810)		0.823
≥65 years	595	64	ref.	-	558	32	0.598 (0.391-0.916)		0.018
Smoking									
No	748	56	ref.	-	774	33	0.628 (0.408-0.967)		0.035
Yes	264	17	ref.	-	235	8	0.658 (0.282-1.533)		0.332
Tipple									
No	853	65	ref.	-	845	38	0.656 (0.439-0.979)		0.039
Yes	159	8	ref.	-	164	3	0.472 (0.124-1.803)		0.272
Hypertension									
No	297	17	ref.	-	259	4	0.321 (0.108-0.957)		0.041
Yes	715	56	ref.	-	750	37	0.704 (0.464-1.068)		0.099
Diabetes									
No	766	59	ref.	-	694	29	0.607 (0.389-0.947)		0.028
Yes	246	14	ref.	-	315	12	0.757 (0.349-1.641)		0.480
Dyslipemia									
No	563	45	ref.	-	334	13	0.365 (0.130-1.024)		0.056
Yes	449	28	ref.	-	675	28	0.782 (0.492-1.243)		0.298
TOAST classification									
LAA	395	29	ref.	-	416	22	0.588 (0.305-1.135)		0.114
SAO	553	34	ref.	-	548	13	0.588 (0.305-1.135)		0.114
Other typing	64	10	ref.	-	45	6	0.588 (0.305-1.135)		0.114

RC≥0.72 mmol/L did not significantly correlate with an elevated risk of all-cause death in males, those aged ≥65, or nondiabetic patients after controlling for age, sex, smoking, drinking, hypertension, and diabetes history (P>0.05 for all) (Table 5).

**Table 5:** Analysis of association between subgroups with different RC levels and risk of all-cause death (adjusted covariates). Note: ref. as the reference group

Factor	RC<0.72 mmol/L				RC≥0.72 mmol/L				
	Number of cases	Number of deaths	HR (95%CI)	P value	Number of cases	Number of deaths	HR (95%CI)		P value
Sex									
Male	658	147	ref.	-	552	79	0.839 (0.626~1.123)		0.238
Female	354	69	ref.	-	457	64	0.737 (0.516~1.053)		0.094
Age									
<65 years	417	39	ref.	-	451	34	0.754 (0.465~1.221)		0.251
≥65 years	595	177	ref.	-	558	109	0.783 (0.607~1.009)		0.059
Smoking									
No	748	172	ref.	-	774	119	0.761 (0.594~0.975)		0.031
Yes	264	44	ref.	-	235	24	1.018 (0.587~1.765)		0.949
Tipple									
No	853	197	ref.	-	845	127	0.752 (0.594~0.953)		0.018
Yes	159	19	ref.	-	164	16	1.870 (0.823~4.249)		0.135
Hypertension									
No	297	52	ref.	-	259	32	1.013 (0.624~1.646)		0.957
Yes	715	164	ref.	-	750	111	0.753 (0.584~0.972)		0.029
Diabetes									
No	766	162	ref.	-	694	94	0.779 (0.596~1.018)		0.068
Yes	246	54	ref.	-	315	49	0.885 (0.578~1.354)		0.573
Dyslipemia									
No	563	119	ref.	-	334	40	0.828 (0.628~1.092)		0.182
Yes	449	97	ref.	-	675	103	0.830 (0.589~1.169)		0.287
TOAST classification									
LAA	395	84	ref.	-	416	71	0.737 (0.516~1.053)		0.094
SAO	553	111	ref.	-	548	59	0.737 (0.516~1.053)		0.094
Other typing	64	21	ref.	-	45	13	0.737 (0.516~1.053)		0.094

There was no significant association between RC≥0.72 mmol/L and an increased risk of death from IS in patients aged ≥65 years, nonsmokers, nondrinkers, nonhypertensive patients, or nondiabetic patients (all P>0.05) (Table 6).

**Table 6:** Analysis of association between subgroups with different RC levels and risk of death from IS (adjusted covariates). Note: ref. as the reference group

Factor	RC<0.72 mmol/L				RC≥0.72 mmol/L				
	Number of cases	Number of deaths	HR (95%CI)	P value	Number of cases	Number of deaths	HR (95%CI)		P value
Sex									
Male	658	51	ref	-	552	26	0.864 (0.520-1.434)		0.572
Female	354	22	ref	-	457	15	0.527 (0.266-1.044)		0.066
Age									
<65 years	417	9	ref	-	451	9	0.879 (0.336-2.302)		0.793
≥ 65 years	595	64	ref	-	558	32	0.675 (0.430-1.060)		0.088
Smoking									
No	748	56	ref	-	774	33	0.661 (0.420-1.042)		0.075
Yes	264	17	ref	-	235	8	1.134 (0.440-2.920)		0.794
Tipple									
No	853	65	ref	-	845	38	0.727 (0.476-1.112)		0.142
Yes	159	8	ref	-	164	3	0.911 (0.176-4.719)		0.912
Hypertension									
No	297	17	ref	-	259	4	0.494 (0.152-1.611)		0.242
Yes	715	56	ref	-	750	37	0.774 (0.499-1.202)		0.254
Diabetes									
No	766	59	ref	-	694	29	0.673(0.421-1.075)		0.097
Yes	246	14	ref	-	315	12	1.016 (0.429-2.409)		0.970
Dyslipemia									
No	563	45	ref	-	334	13	0.801 (0.497-1.291)		0.362
Yes	449	28	ref	-	675	28	0.602 (0.311-1.165)		0.132
TOAST classification									
LAA	395	29	ref	-	416	22	0.527 (0.266-1.044)		0.066
SAO	553	34	ref	-	548	13	0.527 (0.266-1.044)		0.066
Other typing	64	10	ref	-	45	6	0.527 (0.266-1.044)		0.066

### Kaplan-Meier survival curve analysis of patients in the RC group with low water levels and the RC group with high water levels

**Figure 2:**
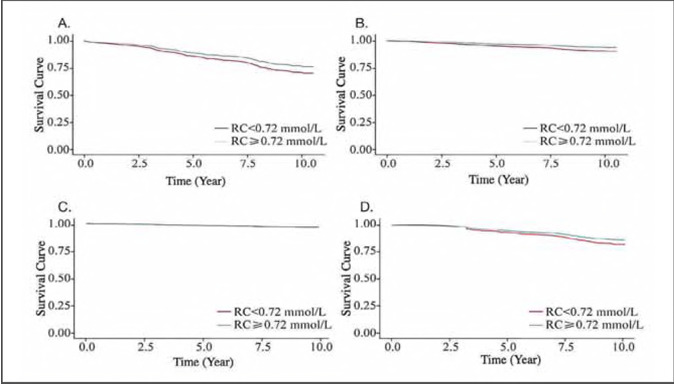
Survival curves of IS patients with different outcomes. A. All-cause death; B. IS-cause death; C. HS-cause death; D. Other-cause death

The median follow-up was 5.52 years. Among the 2021 subjects, 359 all-cause deaths occurred, including 168 stroke deaths (114 IS deaths, 54 hS deaths) and 191 other causes of death (Figure 2). The survival curve showed that the IS patients in the low RC group had a greater survival rate and a lower incidence of IS mortality, all-cause death, and other causes of death than those in the high RC group.

## Discussion

On the basis of a Chinese population cohort of patients with IS, this study investigated the associations between the RC level at admission and the risk of death and IS prognosis [19] [20] [21]. Currently, the medical reference range of the RC IS remains inconclusive; however, according to the RCS regression model established in this study, the level of RC exhibits a significant nonlinear association with the risk of death from prognostic IS and the risk of death from other causes (P<0.001). The curve trend is described, and the median RC level RC=0.72 mmol/L, is determined as the cutoff point [22]. Cox regression analysis revealed that, without adjusting for covariates, higher levels of RC could reduce the incidence of all-cause mortality by 23.5% and the incidence of IS mortality by 36.2% [23]. The Kaplan-Meier survival analysis curve revealed that, compared with those in the high RC group, the IS patients in the low RC group had greater incidences of all-cause death, IS death and death from other causes.

Previous studies [24] [25] [26] have confirmed that RC is a risk factor and risk predictor of atherosclerotic cardiovascular disease (ASCVD) and may replace LDL-C as the main cause of ASCVD. Studies [27] [28] [29] [30] have shown that increased RC levels not only increase aortic stenosis and arterial stiffness but also independently affect elevated blood pressure. Multiple lines of evidence from epidemiological, mechanistic, and genetic studies have shown that RC, as a major factor in the formation of atherosclerotic inflammatory components, can promote the development of atherosclerosis and ischemic heart disease, and that elevated RC levels can be used as early markers of atherosclerotic damage. An evaluation of the general population via CT coronary angiography revealed that RC levels were associated with a significant atherosclerotic burden, which is hypothesised to be the mechanism by which elevated RC leads to the accumulation of cholesterol in the blood vessel intima and blockage of blood vessels, thus accelerating the atherogenic process [31] [33] [32].

With the deepening of research on RC and ASCVD, the relationship between RC and IS has become a topic of increasing interest in recent years. A summary of previous studies revealed that the mechanism by which increasing the RC level promotes IS is through the promotion of atherosclerosis, activation of inflammation, and the induction of thrombosis and other forms. High levels of RC (≥1.5 mmol/L) were associated with an 80% increase in the incidence of IS compared with low levels of RC (<0.5 mmol/L). Long-term cohort studies in the Chinese population have shown that increased variability in RC can lead to an increased risk of IS, suggesting that changes in the risk of IS may not result only from simple increases or decreases in RC levels, which deserves attention [34].

With respect to the effect of RC on the prognosis of IS, a study of genetic variation indicated that a higher baseline RC level (≥0.43 mmol/L) was associated with a 56% increased risk of severe disability and death from IS. A prospective cohort study [35] showed that controlling RC levels to <0.83 mmol/L reduced the recurrence rate of major cardiovascular events by 20% in patients who had already experienced a myocardial infarction or IS. However, the study did not classify myocardial infarction and IS, and it was not possible to determine the exact effect of RC levels on the prognosis of IS. The above studies [36] [37] [38] indicate that the incidence and unfavourable prognosis of IS appear to be associated with the RC level. Given the close relationship between RC and atherosclerosis, it is speculated that the mechanism of action is primarily related to the effect of RC on the progression of atherosclerosis. LAA-type IS is the most common type of IS, and the underlying disease is caused by the rupture of vulnerable plaques in atherosclerotic plaques, which block cerebral arteries and eventually lead to cerebral infarction. This is consistent with the view that IS is also associated with this condition. However, low RC levels at admission were not conducive to patient outcomes. On the basis of the baseline data, the low-level RC group had a greater proportion of men and people older than 65 years, and advanced age was a risk factor for poor stroke outcomes; thus, the low-level RC population had a relatively high risk of death. In the subgroup analysis of this study [39], a higher RC was more significantly associated with a lower risk of all-cause death in elderly, nonsmoking and nondrinking patients, suggesting that the blood lipid status of male patients with RCs<0.72 mmol/L and those ≥65 years of age should be closely monitored, a slightly higher level should be maintained as much as possible, a good lifestyle should be cultivated, and avoiding smoking and alcohol may help reduce the risk of death [40] [41] [42].

In addition, studies [43] [44] [45] have shown that one-third of the total cholesterol (TC) in plasma IS is in the form of an RC. According to Mendelian randomisation studies, residual lipids are heterogeneous and have a dual effect on IS. In this study, a higher baseline RC was found to be associated with a lower risk of death in IS patients, which appears to contradict the conclusions of previous studies on the correlation between RC level and IS prognosis. However, this may also be due to the dual effect of residual lipids on IS, which may be related to the increased risk of IS caused by the higher RC variability mentioned above [46]. The mechanism by which the RC level influences the prognosis of patients with IS is not fully understood, and further studies are needed to investigate the relationship between the RC level and prognosis [47].

In this study, an RC≥0.72 mmol/L at admission was significantly associated with a reduced risk of all cause death and IS-related death in patients with IS, suggesting that assessing patients' RC levels at admission has a clinically guiding effect on the onset of IS and post-discharge interventions. Moreover, this study revealed that the RC level of elderly male IS patients should not be too low, their blood lipid levels should be closely monitored and controlled, and more accurate treatment and nursing measures should be adopted to develop more effective rehabilitation measures [48].

There are still limitations to this study. First, the mechanism by which RC affects the prognosis of IS is not fully understood. Second, there are limitations in the selection of the study cohort, and further attention and research are needed on the differential effects of other geographical regions and different dietary cultures on RC. Finally, the study subjects included in the cohort were patients from various units and departments. In the future, we may consider collecting recent studies from the same source for follow-up, to determine patient prognosis and outcomes, and further verify the conclusions.

The baseline RC level at admission in IS patients was strongly correlated with the predicted death risk, and an RC<0.72 mmol/L at admission in IS patients increased the risk of long-term all-cause death and IS death after discharge. The effect of RC on the prognosis of IS is important for guiding treatment and recovery, and further in-depth studies are needed.

## Dodatak

### Funding

This work was supported by Jinhua Science and Technology Bureau (2022-4-161).

### Availability of data and materials

The datasets generated or analysed during the current study are not publicly available because they contain private information. However, the data are available from the corresponding author upon rea sonable request.

### Authors' contributions

All the authors contributed to editorial changes in the manuscript. All the authors read and approved the final manuscript. All authors have participated sufficiently in the work and agreed to be accountable for all aspects of the work.

### Conflict of interest statement

All the authors declare that they have no conflict of interest in this work.
